# Establishing the pig as a translational animal model for neurodevelopment

**DOI:** 10.1515/tnsci-2025-0369

**Published:** 2025-04-24

**Authors:** Loretta Teresa Sutkus, Zimu Li, Ryan Neil Dilger

**Affiliations:** Neuroscience Program, University of Illinois, Urbana, Illinois, 61801, United States; Department of Animal Sciences, Division of Nutritional Sciences, University of Illinois, Urbana, Illinois, 61801, United States

**Keywords:** translational animal model, domestic pig, neurodevelopment, translating time, prenatal

## Abstract

**Background:**

Within the last few decades, the domestic pig has emerged as an advantageous biomedical animal model due to a vast number of similarities in realms of development and neuroanatomical features. Even so, a major challenge remains in how to translate time between the pig and human. Previously, researchers have developed a Translating Mammalian Time model that estimates the timing of 95 neurodevelopmental events across 9 mammalian species. By identifying the timing of these various events, one can include an additional animal into the model and assign a unique species score to predict the post-conception day (PCD) that other events will occur.

**Objective:**

Our objective was to conduct a comprehensive literature review of pig neurodevelopmental events to enable chronological comparison to other mammalian species, including humans.

**Methods:**

A total of 30 neurodevelopmental events with corresponding PCDs were identified, that were then used to optimize the pig’s species score using grid search and gradient descent approaches.

**Results and Conclusion:**

Across both methods, the same species score of 2.157 was derived with a residual sum of squares of 4260.46. This species score places the domestic pig between the cat (1.808) and the macaque (2.255), thereby reinforcing the translational power of the pig comparable to non-human primates.

## 1 Introduction

Utilizing biomedical animal models is a common practice in the field of neurodevelopment. However, selecting an appropriate animal model requires careful consideration, particularly when studying neurodevelopmental disorders and conditions. Traditionally, researchers have depended on rodent models to investigate development due to a variety of advantages, such as shorter gestational periods, lower costs, and the ease of genetic manipulation [1]. With a tremendous array of transgenic models, mice and rats have been extensively studied in the realm of neurodevelopmental disorders [2]. Despite their numerous advantages, several limitations hinder their applicability to humans. Differences in genetics, behavior, as well as the lissencephalic nature of their brain, make it difficult to replicate neurodevelopmental disease symptoms and conditions in rodents [3]. Therefore, other animal models, such as non-human primates, have been frequently utilized [3]. Non-human primates are highly translatable to humans because of their similarities in social behavior, gestational length, and comparable brain cytoarchitecture [3]. However, many ethical and cost concerns arise when utilizing non-human primates due to the increased physical, psychological, and longitudinal factors that need to be considered. Hence, utilizing alternative, translational large animal models, such as the domestic pig, has become more prevalent.

Over several decades, pigs have been utilized as animal models for nutrition [4], traumatic injury [5,6], neurodegenerative disorders [7], early life insults [8], neurodevelopment [9], as well as neurodevelopmental disorders [10]. The plethora of shared features with humans, rapid growth, and development, as well as the litter-bearing nature of the pig gives a competitive advantage over other animal models. It is well known that the pig brain is gyrencephalic, with well-defined anatomical regions [11,12]. Several pig-specific atlases have also been created and are publicly available that divide the pig brain into both cortical and subcortical regions, making neuroanatomical delineations accessible [12,13]. Additionally, fetal pigs have been historically used in mammalian embryology due to the similarities in embryonic development in relation to fetal stages and shared growth spurts [8,14]. Recently, these embryonic similarities have allowed researchers to model various neurodevelopmental disorders in the pig. For instance, psychiatric conditions such as autism spectrum disorder [15] and intellectual disorders [10], as well as early life brain injuries, such as hypoxic-ischemic encephalopathy [8], have been successfully modeled in the pig. Using a model such as the pig has advantages since early fetal developmental stages [14], neurulation [16], central nervous system development [17], longitudinal postnatal brain growth [18] and myelination [19] have been well characterized, creating a rich foundation for understanding the translational capacity of the domestic pig. Despite its potential, significant gaps remain in solidifying the pig as a translational animal model for neurodevelopment. Most importantly, it is still uncertain how close in proximity the domestic pig is with non-human primates regarding brain development. Establishing the degree of relation between these species is pivotal for data interpretation.

Previous works by Workman et al. [20] and Clancy et al. [21] have been crucial in understanding developmental translation between animal species. This group has created a multiple regression model to predict the timing of neural development across 271 developmental events in 18 mammalian species [20]. Working from a database of 1,010 observations, Workman and colleagues [20] were able to optimize the model with the “quasi-newton” method, providing an excellent resource for developmental translation. Even so, a significant omission in this model is the domestic pig. Hence, our main goal was to incorporate the domestic pig into this model for comparable translation to other biomedical models as well as humans. However, because the Workman et al. [20] model was optimized using a pre-collected database, integrating an additional species presented technical challenges. Addressing this issue required investigation into a previous established version of the model [21] that provided clear accessibility to expansion. In this previous version of the model, the timing of 95 neurodevelopmental events was predicted across 9 mammalian species. Each developmental event was assigned an “event score” and each mammalian species was given a “species score” for comparable translation of timing of the events. Inclusion in the model required identifying the exact post-conception day (PCD) of occurrence for at least one neurodevelopmental event within the model framework [21,22].

Therefore, the main objective of this review was to identify as many porcine-specific neurodevelopmental events as possible to optimize the species score and in doing so create a comprehensive neurodevelopmental timeline from conception to birth in the domestic pig. Within the literature review discussion, each section is divided into neural systems based on the neurodevelopmental events listed in the Clancy et al. [21] model, capturing key milestones across visual, motor, cortical, and limbic systems. This review not only provides a resource for researchers using the pig as a biomedical model, but also solidifies the domestic pig’s relevance in the study of neurodevelopmental processes.

## 2 Methods

### 2.1 Literature search and selection

**Figure 1:**
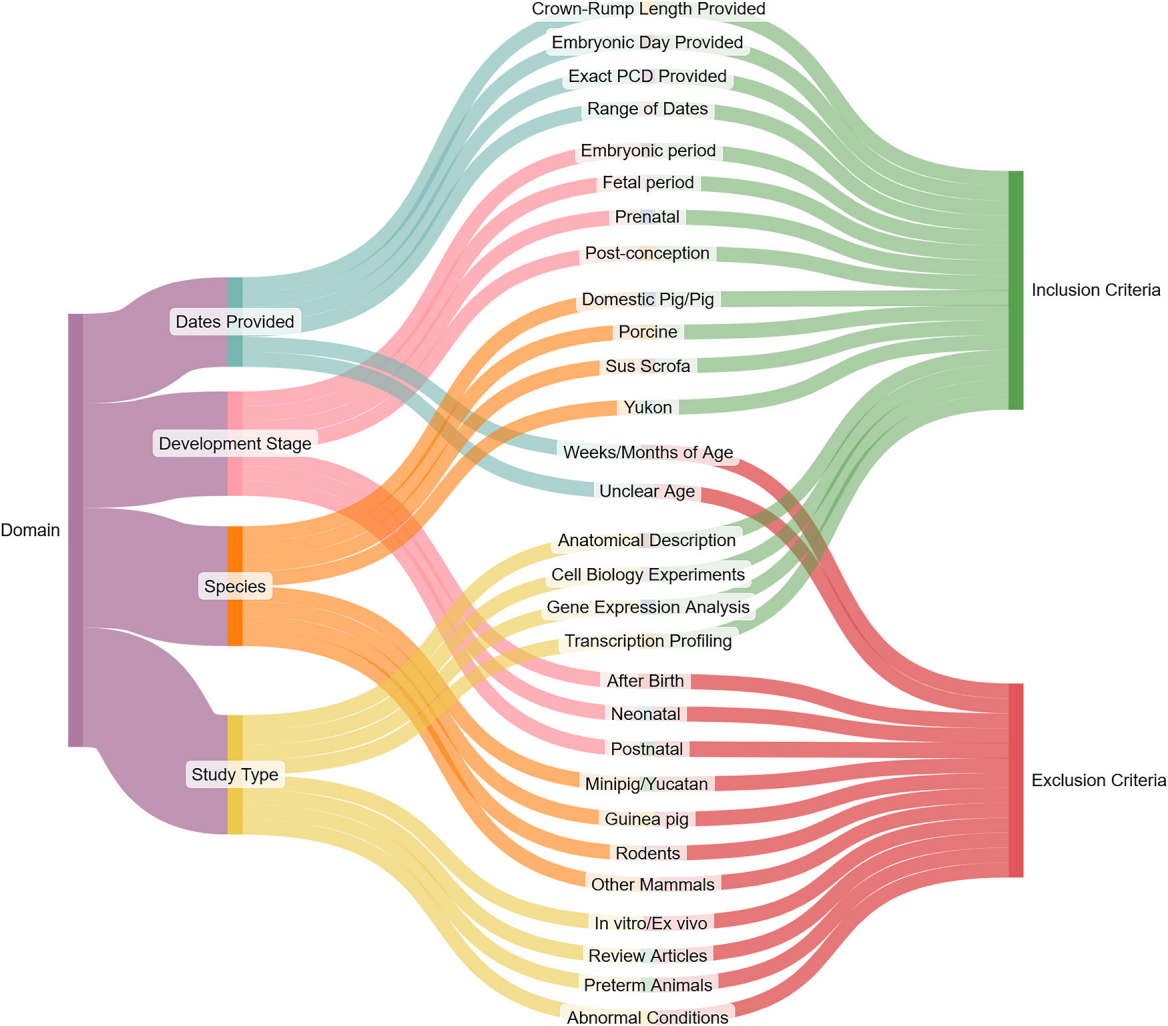
Inclusion and exclusion criteria for literature cited. Literature was selected for the general neurodevelopmental literature review and evaluated across four search domains. Development stage (1) refers to the timing of development that was captured in the article, whether it be prenatal or postnatal growth. Generally, if the literature depicted any portion of the prenatal growth period it was included, any literature that solely focused on development after birth was excluded. Dates provided (2) refers to the actual timing of the neurodevelopmental event. If a clear date, crown-rump length, or range of dates was provided, it was included into the literature review. The species criteria (3) excluded animal species that did not belong to the domestic pig or related breeds. Finally, the study type domain (4) refers to the type of experiment or assessment used to identify the neurodevelopmental event. The goal was to obtain concrete information (i.e., neurogenesis, cellular proliferation, anatomical depictions, gene expression, etc.) on the normal growth of a pig embryo. Abbreviations: PCD, post-conception day.

Multiple literature search engines were utilized when searching for neurodevelopmental events such as, but not limited to, Google Scholar, Elicit, Europe PMC, PubMed, Semantic Scholar, and Science Direct. Based on a manual review of the title and abstract, articles were immediately excluded based on a series of inclusion/exclusion criteria depicted in Figure 1.

This literature review was solely focused on evaluating neurodevelopment in domestic pigs and related breeds. Thus, literature pertaining to animal models such as minipigs, rodents, primates, and guinea pigs were excluded. Additionally, prenatal development was of particular focus and hence any studies with only postnatal or mature animals were excluded. Furthermore, given that the main interest of this review was to capture a typical timeline of gestational neurodevelopment in the pig, any developmental reviews that focused on neurological diseases, preterm animals, deficiency models, or other abnormal conditions were also excluded.

Furthermore, specific criteria were assessed when selecting which neurodevelopmental events to include. In general, if the literature listed a neurodevelopmental event in one of the regions of interest listed by Clancy et al. [21], with an associated PCD, the event was included in the overall literature review. To be considered for inclusion into the model by Clancy and colleagues [21], the selected event had to match one of the 95 that they documented. Exact verbiage of each event was taken into consideration when assessing its inclusion in the model, following the guidelines outlined in Table 1. Additionally, in cases where a crown-rump length for pigs was reported instead of an exact post-conception date, the PCD was estimated using the equation provided by Ullrey and colleagues [23].

**Table 1:** Inclusion/exclusion criteria for literature review verbiage of neurodevelopmental events^1^

Neural event	Inclusion criteria			Exclusion criteria
	Tier 1	Tier 2	Tier 3	
Region appears or starts…	“Clear distinction”	“Early phase”	Neurogenesis, differentiation, or proliferation “begins”	Region “is present”
	“Production begins”	“First element”	“Emergence of cells”	Region is “well developed”
	Neurogenesis “likely to start”	“Region will develop into”	Region first “appears”	“Early recognition”
	“Fibers present”	“Beginning” of region	“First trace” or “indication”	“Hardly identifiable”
	Region “recognizable”		“Present for the first time”	“Not apparent”
	Region “detectable”		“Appearance”	
Region peak…	“Well developed”	“Mostly complete”	“Adult labeling”	“Growth completed”
	“Distinct layers”	“Neurogenesis ends” or “complete”	“Adult patterning”	“Differentiation completed”
	“Growth begins to slow down”	“Appears complete”	“Proliferation peaks”	Region is “mature”
		“Almost normal”	“Region peaks”	“Definite” region present
		“Full differentiation”		Sub- regions or fields “distinct”
		“Well defined”		
Region ends…	Region “completed”	“Activity subsides”	Neurogenesis, formation, differentiation, or cell proliferation “Largely complete” or “completed	“Growth slows”
	“Immature cells still present”	“Well developed”	“Neurogenesis ends”	Growth “ends” or “completed”
	“Young adult” cells	Neurogenesis “likely to end”	“Full differentiation”	Region “is fully present”
			“Cell maturation”	“Distinct layers”
				Region is “mature”
				“Definite” region present
				Sub- regions or fields “distinct”

^1^This table depicts how various events were selected for inclusion into the final list of neurodevelopmental events that would be used for the pig species score optimization. Verbiage was taken into consideration when determining how close each event pertained to the events listed by Clancy et al. [21]. Within the inclusion bracket, there were 3 tiers that would determine if the region would be assigned a CL of 1, 2, or 3. The three tiers correspond to the three CLs where tier 1 is the most ambiguous verbiage, while tier 3 is the most clear and defined verbiage.

### 2.2 Assigning confidence levels (CLs)

After identifying as many neurodevelopmental events as possible, the next step was to refine the selected events based on the accuracy of the methods used to classify the event. Each event was assigned a CL from 1 to 3, where 1 was the lowest confidence and 3 was the highest. The criteria for assigning these CLs are depicted in Tables 1 and 2. For example, in cases where a range of days was provided instead of an exact PCD, the event was assigned a CL of 1. In cases where a brain region was stated to “appear” or there was the “first trace” of the region without an assessment of cell types or indication of proliferation activity, the event was assigned a level of 2. The events with a CL of 3 had a clearly assigned post-conception date, used almost the exact verbiage as Clancy and colleagues [21], and explicitly mentioned neurogenesis or cellular proliferation. 

**Table 2:** Neurodevelopmental event CL assignment

CL		
1	2	3
Week of fetal development or range of crown-rump length provided – needed to calculate average	Exact fetal days or crown-rump length provided	Exact fetal days or crown-rump length provided
Verbiage for event falls in Tier 1	Verbiage for event falls in Tier 2	Verbiage for event falls in Tier 3
Ambiguity in the referenced event without clear evidence^1^	Verbiage for event falls in Tier 1 with clear evidence^1^	Verbiage for event falls in Tier 2 with clear evidence^1^
Specific event not directly stated, but implied	Ambiguity in the event that it is referencing	Event from Clancy et al. [21] directly referenced
General region referenced more broadly	Multiple events/regions mentioned at the same time	Clear distinction of regions discussed

^1^Clear evidence refers to direct quantification of neurogenesis, cell proliferation/differentiation, reference to gene expression of related cells, or utilizing relevant cell markers.

### 2.3 Species score calculation

Next we utilized the established equation created by Clancy and colleagues [21] to position the pig on the neurodevelopmental translational timeline using the following equation:

(1) \[Y=\mathrm{ln}({\mathrm{PCD}}-4.42),]\]

where \[Y]\] is the overall translational function that combines the event and species scores for a specific species, PCD is the post-conception day at which a specific neurodevelopmental event occurs, and 4.42 is a predetermined constant derived by Finlay and Darlington [24]. This constant represents the average duration in days required for early developmental processes such as implantation, blastulation, and differentiation to occur, which are relatively consistent neurodevelopmental events across eutherian mammals.

Since *Y* is the sum of the species and event scores:

(2) \[Y={\mathrm{Species\; Score}}+{\mathrm{Event\; Score}},]\]

the equation can be reparametrized to solve for the species score, given an individual event:

(3) \[{\mathrm{Species\; Score}}=\mathrm{ln}({\mathrm{PCD}}-4.42)-{\mathrm{Event\; Score}}.]\]

This equation was applied to all selected pig neurodevelopmental events identified in the literature using the observed PCD along with the corresponding event score from Clancy and colleagues [21]. This process generated a range of species scores for the domestic pig, reflecting variability across calculated species scores given to different events. This range served as the initial species score estimate for the optimization process described in Section 2.4.

### 2.4 Species score optimization

The optimization of the species score involved minimizing the residual sum of squares (RSS) between observed and predicted PCD values for neurodevelopmental events. We approached this optimization task with two different methods – grid search and gradient descent. Using both methods ensured that the final species score was accurate and consistent across different calculation methods.

For both methods, species score optimizations were repeated three separate times using a different dataset that included events with varying CLs. The first calculation included all neurodevelopmental events found in the literature \[({\mathrm{CL}}\left\ge 1)]\] . The second repetition included neurodevelopmental events with a CL higher than 1 \[({\mathrm{CL}}\left\ge 2)]\] . The last repetition included only events that we were most confident in \[({\mathrm{CL}}\left\ge 3)]\] .

The optimization algorithms were implemented using Python 3.11.9 and using libraries including NumPy [25] for numerical computations, Pandas [26,27] for data handling, and Matplotlib [28] visualizing the loss function and monitoring convergence during gradient descent.

#### 2.4.1 Calculation of predicted PCD and loss

To compute the predicted PCD for each neurodevelopmental event, the following equation was derived from equation (3):

(4) \[{\mathrm{PCD}}=\hspace{1em}{{\mathrm{e}}}^{({\mathrm{Event\; Score}}+{\mathrm{Species\; Score}})}+4.42.]\]

This equation allowed for the calculation of the predicted PCD using the event score and a candidate species score.

To evaluate how well a candidate species score explains the observed data, the RSS was used as the loss function:

(5) \[L=\mathop{\sum }\limits_{i=1}^{n}{({\mathrm{PC}}{{\mathrm{D}}}_{\text{obs},i}-{\mathrm{PC}}{{\mathrm{D}}}_{\text{pred},i}(\text{Species Score}))}^{2},]\]

where \[{{\mathrm{PCD}}}_{{\mathrm{obs}},i}]\] represents the observed PCD that is reported for the \[i{\mathrm{th}}]\] event from the literature and \[{{\mathrm{PCD}}}_{{\mathrm{pred}},i}]\] represents the predicted PCD for the \[i{\mathrm{th}}]\] event using the candidate species score. Here the RSS quantifies the difference between observed and predicted PCD values. A smaller RSS indicates a closer match between observed and predicted values, and therefore a more optimized candidate species score.

#### 2.4.2 Grid search

Grid search systematically explores the predefined range of species scores from Section 2.3, evaluating each candidate species score to identify the one that minimizes the loss as defined in equation (5). This method was described by Bergstra and Bengio [29] as a straightforward approach for parameter optimization in models where exhaustive evaluation of possibilities is computationally feasible. The optimization process is described as follows:

(6) \[\text{Species}{\text{Score}}_{\text{optimal}}={\mathrm{\arg }}\mathop{\min }\limits_{\text{S}\left\in \left\{{S}_{{\mathrm{c}}}\right\}}\mathop{\sum }\limits_{i=1}^{n}{({{\mathrm{PCD}}}_{\text{obs},i}-{{\mathrm{PCD}}}_{\text{pred},i}(\text{Species Score}))}^{2},]\]

where \[S]\] represents the candidate species score and *S*_c_ represents the range of candidate species scores, which were systematically explored.

The candidate species score range was determined from Section 2.3 with a step size of 0.001. For each \[{S}_{{\mathrm{c}}}]\] , the predicted PCD was calculated using equation (4), and loss was computed using equation (5). As described in equation (6), the optimization process iteratively computed the loss for each \[{S}_{c}]\] , and the optimal species score was selected as the one with the least loss.

#### 2.4.3 Gradient descent

Gradient descent was implemented separately in optimizing the species score by minimizing the loss defined as equation (5). As described by Baldi [30], this method achieves optimization by iteratively updating the target parameters in the direction of the steepest descent of the loss function. The gradient of our loss with respect to the species score was calculated as

(7) \[\frac{\partial L}{\partial \text{Species Score}}=\mathop{\sum }\limits_{i=1}^{n}{[}-2\cdot ({\text{PCD}}_{{\mathrm{obs}},i}-{\text{PCD}}_{{\mathrm{pred}},i})\cdot {{\mathrm{e}}}^{(\text{Event}{\text{Score}}_{i}+\text{Species Score})}].]\]

Using this gradient, the species score was updated at each iteration according to the following equation:

(8) \[{\mathrm{Updated\; Species\; Score}}={\mathrm{Current\; Species\; Score}}\hspace{10.5em}-\alpha \cdot \frac{\partial L}{\partial \text{Species Score}},]\]

where learning rate ( \[\alpha ]\] ) is set to 1 × 10^−5^, representing the step size of each update to the species score. This learning rate prevents large updates that could overshoot the minimum, while still allowing gradual convergence to the optimal value in reasonable time. The algorithm was tested for convergence at each iteration and terminated when either of the following criteria were met:

(9) \[|{\mathrm{Updated\; Species\; Score}}-{\mathrm{Current\; Species\; Score}}|< \text{tolerance or}t\ge \max \text{iterations},]\]

where the tolerance was set to 1 × 10^−6^, \[t]\] represented the current number of iterations, and the max iterations was set to 10,000.

Figure 3 illustrates the gradient descent process, showing how the gradient of the loss function determined the direction and magnitude of updates to the species score. The optimizer made iterative adjustments along the green arrows that guided the algorithm toward minimizing loss and achieving convergence at the optimal species score (i.e., minimum value) represented by the red dot.

## 3 Results and discussion

### 3.1 Literature review

**Figure 2:**
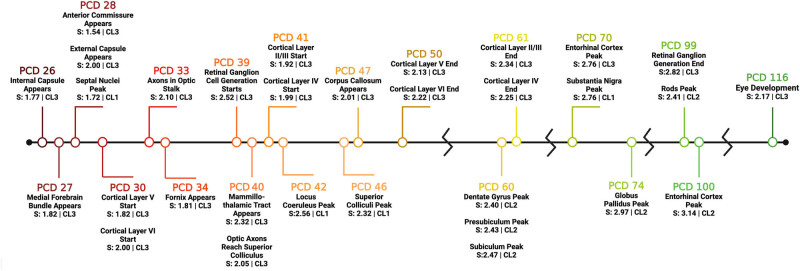
Pig neurodevelopmental timeline. This figure depicts the accumulation of all the neurodevelopmental events selected for pig species score optimization in one comprehensive timeline. Each event has a corresponding PCD, species score (*S*), and CL. Each PCD depicted in this timeline is the date derived from the literature. The species score for each event was calculated individually using the depicted PCD and associated event score from the translating time model. CLs were assigned based on the criteria in Tables 1 and 2.

The following literature review provides a comprehensive summary of pig gestational development, spanning from week 3 of gestation through birth at 16 weeks. This section is divided into four subsections covering four distinct brain systems: the visual system (Section 3.1.1), motor system (Section 3.1.2), cortical organization and connections (Section 3.1.3), and limbic system (Section 3.1.4). Each subsection begins with a brief overview of the relevant anatomy followed by a detailed timeline of gestational development in that specific system. The cited literature spans nearly a century, from one of the earliest anatomical documentations of the pig embryo in 1932 [31] to more recent gene expression profiling in 2021 [32]. By covering this large expanse of literature, a wide range of neurodevelopmental events in the developing pig was captured. The pig-specific neurodevelopmental events described here were the basis of the neurodevelopmental timeline presented in Figure 2 and selected events are denoted with a symbol (*).

#### 3.1.1 Visual system

The visual system is one of the first sensory systems to develop in the mammal. Apart from eye formation during gastrulation, which is relatively similar across species, the development of the retina is the most well-documented visual process to occur in the pig [33].

##### 3.1.1.1 Relevant anatomy and overview

Retinal cellular development is characterized by relatively asymmetric cellular division. Initially, the retina size is determined by the number of cell cycles that retinal progenitor cells undergo [34]. Proliferating cells transition into ganglion, horizontal, photoreceptor, bipolar, amacrine, or Müller glial cells each with specific retinal functions [32].

From the outermost layer inward, photoreceptor cells are the first cells to respond to light and reside in the photoreceptor outer segment and outer nuclear layer of the retina [35]. Photoreceptor cells are divided into rods, which are highly sensitive to light, and cones, which require bright conditions to function and are responsible for color vision [36]. In the outer plexiform layer, horizontal cells play a crucial inhibitory role, modulating brightness and enhancing images projected onto the retina [35]. Next within the inner nuclear layer bipolar cells act as essential relays that connect photoreceptor cells to ganglion cells, respond to light, and are important for color vision [37]. Additionally, Müller glial cells also reside within this layer and are fundamental for structural and metabolic functions within the retina [38]. Amacrine cells are also found in the inner nuclear layer and, like bipolar cells, synapse with ganglion cells in the inner plexiform layer [35]. There are around 30 different types of amacrine cells, that primarily function as interneurons for bipolar and ganglion cells [39]. Finally, in the ganglion cell layer, ganglion cells, receive input from bipolar and amacrine cells and transmit this information to the brain through the optic nerve [40].

From the optic nerve, ganglion cell axons travel through the optic chiasm and tract until they synapse in the lateral geniculate nuclei of the thalamus, an important visual relay station [41]. Medial to the lateral geniculate nuclei are the superior colliculi, which are separated into deep and superficial layers, process visual stimuli, and help orient eye and head movements. The superficial layers receive direct input from the retina and are specialized for visual information processing [41]. These regions represent only a subset of the visual system, which is covered in detail in the review by Prasad and Galleta [41].

##### 3.1.1.2 Early gestation visual development in the pig

At PCD 27, division of the primitive neural retina occurs [33] concurrently with proliferation of the alar plates within the superior colliculus [31]. The primitive neural retina divides into a thick multi-layered neuroblastic layer, which will give rise to the ganglion cell layer and inner and outer nuclear layers, and a thin non-nucleated marginal layer, or the future nerve fiber layer [33]. At PCD 28, a well-developed optic tract and extensive supraoptic commissure are present, with lateral fibers from the supraoptic commissure entering the optic tract [42]. Soon afterwards, on PCD 32, the mantle layer of the superior colliculi differentiates by throwing off a superficial shell of densely packed cells [31]. This shell of densely packed cells is the future stratum griseum, a sensory layer within the superior colliculus that responds to bilateral retinal inputs from visual stimuli [43]. At the same time, the oculomotor nuclei appear as a simple clump of cells that begin dividing into medial and lateral parts [31]. Between PCD 33 and 40, the primitive nerve fiber layer undergoes differentiation from the marginal layer, also known as the ganglion cell layer [33]. De Schaepdrijver and colleagues [33] described this ganglion cell layer as containing axons traceable to the optic nerve*. It is possible that De Schaepdrijver and colleagues [33] are referring to the process of retinal ganglion cell navigation toward the brain, where they grow toward the optic stalk, change course at the optic disk, and pass by the developing optic nerve cells before continuing onwards [44]. Furthermore, during PCD 33–40, Ghosh and Arnér [45] identified the emergence of Müller cells, ganglion cells, and immature photoreceptors within the primitive plexiform and both inner and outer parts of the neuroblastic cell layer on PCD 39*. At this stage, the neuroblastic cell layer consists of multiple rows of undifferentiated cells with an inner marginal zone that will remain present until PCD 49 [45]. Between PCD 40 and 46, the ganglionic cells move inwards while the Müller cells move outwards in a posterior-peripheral direction [33]. Subsequently, between PCD 46 and 55, retinal layer stratification begins, resulting in the formation of the definitive inner plexiform layer and posterior development of a single ganglionic cell layer [33].

Elsewhere in the developing pig brain, by PCD 34, the optic radiation tract, which carries visual information from the retina to the visual cortex, is clearly traceable to the superior colliculus [31]. Soon after, optic fibers then invade the superior colliculus at PCD 40, and the distinct layers of the superior colliculus are apparent at PCD 46* [31]. Concurrently with layer distinction, the Edinger–Westphal nucleus, a region part of the oculomotor cortex that is important for pupillary constriction and lens accommodation [41], appears as a collection of small cells [31].

##### 3.1.1.3 Photoreceptor development in the pig

Rod and cone development in pigs progresses around the midpoint of gestation. On PCD 50, the outer two rows of the retina are populated by post-mitotic cone progenitors and retinal progenitor cells express proliferating cell nuclear antigen, signifying active proliferation [46]. Within the neuroblastic layer, PAX6 expression diminishes between PCD 56 and 65, indicating that cells are becoming post-mitotic precursors, in an outer-to-inner progression [46]. For example, during this time, post-mitotic neural retinal rod precursors appear [46]. Additionally, on PCD 60, Ghosh and Arnér [45] reported the production of rhodopsin in photoreceptor cells, signifying the beginning of rod differentiation. This is contrary to the study by Wang and colleagues [46], who observed the first evidence of rhodopsin later at PCD 85. Even so, at PCD 65, neural retina leucine zipper (NRL) expression is induced in the outer retina, marking rod precursor cells, whereas RCVRN+ expression is evident on inner retinal cells, marking rods and cones [46]. Between PCD 67 and 77, the inner nuclear and outer nuclear layers form, mitotic activity halts, and rods, cones, and amacrine cells begin forming [33]. Meanwhile, at PCD 70, the amount of recoverin labeled photoreceptors increases in the retina until the outer nuclear layer (where rods and cones reside) is formed, the outer plexiform layer is officially present, and calbindin labeled horizontal cells as well as parvalbumin labeled AII amacrine cells first appear [45]. At PCD 85, the outer and inner nuclear layers of the neuroblastic layer finally separate. ISRL1 expression diminishes in the outer two rows of the retina, and rods and cones are distinctly evident in the outer nuclear layer. At this stage, L/M opsin expression is fully segregated from rod nuclei, an indication of distinct cones [46]. On the contrary, De Schaepdrijver et al. [33] observed apparent prospective rods and cones between PCD 86 and 99, possibly due to their use of crown-rump length as an indicator of age. By PCD 99, adult labeling patterns for rods, horizontal cells, AII amacrine cells, and ganglion cells are observed, signifying potential peaks in proliferation* [45]. Rods display an almost normal rhodopsin distribution, a marker of adult rod rhodopsin expression, while cone differentiation begins with the production of cone transducin* [45]. Ganglion cell maturation and progressive thickening of the nerve fiber layer and inner plexiform layer in the retina are largely completed, while rod bipolar cells emerge in the inner nuclear layer [45]. By PCD 106, about 8–10 days before birth, RCVRN expression expands to all cells in the outer nuclear layer [46]. With this level of retinal maturity, piglets are born with open eyelids, typically between PCD 114 and 116* [47].

#### 3.1.2 Motor system

Similar to the visual system, the motor system is also an early-developing system in the pig. Domestic pigs are classified as a more precocial species, meaning their locomotor capabilities are well-developed at birth [48]. By the time of birth, pigs demonstrate high static balance performance and relatively complete sensorimotor control [48]. This implies that a large portion of their motor development occurs during gestation.

##### 3.1.2.1 Relevant anatomy and overview

During embryonic development, the central nervous system is divided into several continuous areas that later develop into distinct brain regions. The diencephalon, the most rostral part of the brainstem, comprises primarily the thalamus and hypothalamus [49]. The mesencephalon, or the midbrain, consists of the cerebral peduncles, substantia nigra, mesencephalic tegmentum, and inferior/superior colliculi [49]. The metencephalon includes the cerebellum and pons regions, while the myelencephalon corresponds to the medulla oblongata [49].

Beginning with the most rostral area, the diencephalon is a key regulatory and relay center of the brain. The diencephalon is made up of the thalamus, which acts as a major relay center for motor and limbic processes, and the hypothalamus, which regulates homeostasis [50]. One of the major connections of the thalamus is with the basal ganglia, a collection of input, output, and intrinsic subcortical nuclei, that are involved in motor control, executive functions/behaviors, and emotions [51]. Input nuclei, such as the caudate nucleus, nucleus accumbens, and putamen, receive signals from various sources, while output nuclei, such as the globus pallidus internal segment and substantia nigra pars reticulata, transmit processed information to the thalamus [51]. Meanwhile, the intrinsic nuclei including the external segment of the globus pallidus, subthalamic nucleus, and substantia nigra pars compacta facilitate communication within the basal ganglia [51]. Through this system of nuclei, the basal ganglia have a complex network of inhibitory, excitatory, and dopaminergic pathways that simultaneously activate and inhibit various processes [50].

Moving more caudal lies the mesencephalon, or midbrain, area. The cerebral peduncles, a collection of important nerve fiber tracts, pass through the mesencephalon and connect the cortex to the spinal cord enabling direct muscle control [49]. One of the largest nuclei within this area is the substantia nigra, an essential region for regulating movements [49]. Next to the substantia nigra, is the mesencephalic tegmentum, which includes a large array of nuclei as well as multiple fiber tracts, such as the red nucleus, periaqueductal gray, medial longitudinal fasciculus, and ventral tegmental areas [49,52,53]. These regions are pivotal for motor control, defensive responses, eye movements, and dopamine release, respectively [52,53,54].

Further down the brainstem is the metencephalon, an area pivotal for coordinating movements and maintaining balance [50]. The cerebellum is the key component of this area, divided by deep fissures into three lobes – anterior, posterior, and flocculonodular lobes – which regulate movements and balance [55]. Ventral to the cerebellum sits the pons, an important region that relays information between the cortex and cerebellum [50]. Key cranial nerve nuclei within the pons include the locus coeruleus, which produces norepinephrine, and abducens nuclei, which coordinate eye movements. [53,56]. More caudally sits the myelencephalon or medulla oblongata, the main connection between the brain and spinal cord and a pivotal regulatory area for the cardio-respiratory system [50]. Numerous cortical motor areas and higher order association areas are important for motor function, many of which exceed the scope of the current review and are addressed by Daly and colleagues [50].

##### 3.1.2.2 Week 3 of gestation in the pig

At 3 weeks of gestation in the domestic pig, on PCD 21, the cerebellar primordium, or the earliest identifiable cells of the cerebellum, become distinguishable [57]. Caudal to the level of the primordium, within the metencephalon, a few tyrosine hydroxylase immunoreactive (TH-i.r.) cells are visible, which denote neurons that express an enzyme important for the biosynthesis of catecholamines, such as dopamine and epinephrine [58]. This area with observable TH-i.r. cells signifies the future locus coeruleus [57]. Additionally, at the same time, a few isolated TH-i.r. cells are present in the roof of the mesencephalon and within the ventral mesencephalon, potentially marking early cells of the future substantia nigra [57]. In the developing mesencephalic tegmentum, the walls of the caudal and rostral mesencephalic colliculi are only a few cells thick, and the hypothalamus becomes distinguishable at this time [57]. Soon after at PCD 24, a general supraoptic commissure is present [42]. At PCD 25, several events occur simultaneously in the cerebellum as well as in the motor nucleus. Caudal to the midbrain, a commissure is observed in the roof of the fourth ventricle, and along this commissure, the bilateral halves of the cerebellum begin fusing [59]. Additionally, a furrow divides the cerebellum into bilateral pieces, representing the beginnings of the flocculonodular lobe and the corpus cerebelli [59]. Dorsal to the medial longitudinal fasciculus within the mesencephalon, the medial motor nucleus gives rise to all the motor fibers destined for the trigeminal, facial, glossopharyngeal, vagus, and accessory nerves [60]. Between PCDs 25 and 26, cells migrate outward from the medial motor nucleus, collecting in the ventrolateral part of the medulla to form the facial nucleus and scattered nucleus ambiguus, two areas of motor nerves responsible for facial movements and swallowing, respectively [60,61].

By PCD 27, the corpus cerebelli are noticeably thicker and cerebellum fusion has extended caudally [59]. Additionally, around this time abducens fibers fan out laterally and begin moving ventrolaterally until PCD 27–30, when the migrating cells collect to form the central accessory abducens nucleus [60]. Within the roof of the midbrain, two dorsolateral bulges mark the site of the developing inferior colliculi [42]. During this time at PCD 28, several concurrent events occur in the developing hypothalamus, midbrain, and medulla. In the diencephalon area, an extensive supraoptic commissure spreads transversely across the hypothalamic tube while most medial fibers enter the central gray and central tegmental fasciculus [42]. Within the midbrain, a definite tectospinal tract emerges, cells and fibers of the trigeminal nerve establish a primary afferent pathway, and the lateral funiculus of the spinal cord establishes a second afferent pathway through the midbrain [31]. At this stage, the medulla is more developed compared with the midbrain, with direct and indirect sensory pathways into the cerebellum and a rising small nucleocerebellar tract [31]. Additionally, the medial forebrain bundle, a major neural pathway through the hypothalamus, first appears* [42]. At PCD 29, the beginnings of the cells that populate the caudate nucleus emerge on the medial side of the internal capsule [42].

##### 3.1.2.3 Weeks 4–5 of gestation in the pig

During the fourth week of gestation, at PCD 30, a large group of TH-i.r stained cells emerge orthogonally near the mesencephalic flexure [57]. More rostral to this group, the ascending mesotelencephalic tract begins to form, and a periaqueductal area is discerned near the mesencephalic floor [57]. At this stage, within the locus coeruleus, distinct and well-packed cell groups are observed, which resemble A4 and A6 cell groups, two noradrenergic nuclei within the periventricular gray area [57,62]. Additionally, TH-i.r. cells are observed to be individually scattered throughout the hypothalamus area [57]. Furthermore, at PCD 30, several regions part of the basal ganglia are also developing. The first trace of the globus pallidus appears as a basal nucleus and the striatal area has uniform cytology with observable small dark nuclei, larger lighter nuclei, glioneurocytes, and glial cells [42]. Darker nuclei congregate near the caudate nucleus area, whereas lighter nuclei are prevalent in the neo-cortical area [42]. From PCD 30–37, the caudate nucleus remains as a dormant layer of cells, whereas the nearby ependymal layer of cells proliferates into a thick layer [42]. At PCD 32, large motor cells appear between the oculomotor nerve and habenulo-interpeduncular tract, marking the beginning of differentiation within the red nucleus, which becomes an even more definitive collection of large axons and dendritic stubs by PCD 34 [31]. Furthermore, at PCD 32, the first traces of the subthalamic nucleus arise from a cell column extending from the inner portion of the cerebral peduncle to the mammillary nuclei [42]. The upper end of this cell column becomes the nucleus of Meynert’s commissure, a white matter tract near the optic chiasm, while the lower end becomes the supraoptic nucleus [42]. Additionally, the inferior colliculi also become distinguishable at this point [42]. By PCD 34, most of the hypothalamic region is present except the mammillothalamic tract, the cerebral peduncle first appears, and the medial forebrain bundle extends a branch tract into the pons [42]. Within the medulla, beginnings of the pons region, a cochlear nucleus and trapezoid body are evident [31]. Nearby, the pyramidal tract establishes itself up to the pons region [31].

At PCD 37, the flocculus nodulus, a lobe within the cerebellum important for regulating the vestibular system for balance [55] is distinguishable. At this time, a cap of giant cells covers the caudal pole of the red nucleus [31,59]. The cerebral peduncle has also advanced as far as the pons and beneath this, the first trace of the substantia nigra becomes apparent as a collection of cells [42]. Within the basal ganglia, the globus pallidus remains distinctly separate from the basal nucleus [42]. By PCD 38, migratory cells in the dorsal column of the medulla oblongata become apparent and terminate in the area of the developing pons [59]. At PCD 40, the flocculonodular lobe, as well as the nearby posterolateral fissure are both prominent [59]. Meanwhile, throughout the cerebellum a non-neural aromatic l-amino acid decarboxylase (AADC) isoform is present, and the Purkinje cell layer becomes AADC-positive from this point onwards, marking the synthesis of serotonin and dopamine neurotransmitters [63]. The dentatorubral fibers, which connect the dentate nucleus of the cerebellum to the red nucleus, can now be traced into the red nucleus [31]. In the midbrain, the chief centers and tracts are all present, including the mammillothalamic tract in the hypothalamus* [31].

##### 3.1.2.4 Weeks 6–7 of gestation in the pig

At around 6 weeks into gestation, several cell populations begin differentiating throughout the mesencephalic tegmentum. Dorsal and lateral cell groups exhibit clear mature cell bodies, while the ventral and medial groups are packed with rounded or oval immature cell bodies [57]. A similar cell topography is observed within the periaqueductal gray, where more differentiated cells reside laterally and dorsally, and densely packed undifferentiated and immature cells are found ventromedially [57]. The ventral tegmentum area still contains mostly immature TH-i.r. cells and processes at this time [57]. Lateral to this area, clusters of immature TH-i.r. cells are observed in the substantia nigra medially, whereas laterally well-defined cell bodies and processes are evident [57]. Additionally, at this stage, the A4 and A6 cell groups within the locus coeruleus are now well-differentiated exhibiting distinct processes* [57]. Concurrently, the hypothalamus now has a higher density of mature TH-i.r. cell populations [57]. By PCD 46, a lateral-to-medial gradient of large to small cells is apparent in the substantia nigra, with small cells collecting into a loose nucleus [31,42]. Additionally, the thick ependymal cell layer near the caudate nucleus begins to subside [42]. By PCD 49, multiple fissures such as the posterolateral, primary, and posterior superior fissures become more prominent in the cerebellum [59]. The corpus cerebelli become clearly divided into anterior and posterior lobes [59]. At PCD 52, fibers from the pyramidal tract now extend into the substantia nigra, which is populated by medium sized cells [31]. Additionally, the caudate nucleus changes in structure as the subsiding ependymal mass creates a vacated space, which is subsequently filled by an expanded caudate nuclear stratum [42]. The ependymal mass becomes a narrow layer that covers the caudate nucleus.

##### 3.1.2.5 Weeks 9–10 of gestation in the pig

By PCD 67, one of the motor processing regions of the cerebellum, sub-lobule VIIB, is divided by a shallow sulcus to form three folia [59]. Soon after, at PCD 68, the cells within the subthalamic nucleus resemble those seen in a young adult pig [42]. By PCD 71, a furrow becomes visible on the ventral surface of the cerebellum and the vermian lobules exhibit adult-like patterning in the anterior lobe by PCD 75 [59]. Around the same time, the architectural patterning of the mesencephalon resembles that of adult pigs [57]. Within the mesencephalic tegmentum, a large TH-i.r. cell group forms in the retrorubral field, an area important for assessing threat and aversive outcomes [57]. Clusters of immature cells remain in several areas like the ventral tegmental area, the medial portion of the substantia nigra, raphe nuclei, and subependymal periaqueductal area [57]. However, in the ventral tegmental area, well-differentiated multipolar cells are present, resembling those found in the substantia nigra [57]. At this point, the substantia nigra area is clearly divided into three distinct regions: the pars compacta, the pars lateralis, and pars reticulate [57]. Additionally, by PCD 74, the globus pallidus reaches full differentiation and is divided by scattered fasciculi of the internal capsule* [42].

#### 3.1.3 Cortical organization and connections

Across mammalian species, the cerebral cortex is divided into gray matter and white matter. Gray matter consists of neuronal cell bodies, dendrites, axons, and glial cells, while white matter is primarily bundles of axons that are covered in a myelin sheath, which gives it its distinct white color [64]. The gray matter of the cerebral cortex is further divided into six distinct cortical layers whereas the white matter is organized into a complicated system of fasciculi [65,66].

##### 3.1.3.1 Relevant anatomy and overview

The distinct cortical layers are labeled Layer 1 through Layer 6, corresponding to the most superficial to the deepest layer, respectively [66]. The most superficial layers, Layers 1 and 2, are primarily packed with dendrites and cells that process deeper cortical inputs [66,67]. Layers 3 and 4 are central cortical layers that transport excitatory and inhibitory cortical outputs and process sensory input, respectively [67]. The deepest layers, Layers 5 and 6, contain pyramidal cells that send signals to regions outside of the cortex [67].

On the other hand, white matter is organized into various fiber pathways throughout the brain, essential for efficient communication [65]. Among these tracts, the corpus callosum and anterior commissure are crucial pathways that connect and link various neural circuits between the left and right hemispheres [68,69]. Additionally, the internal and external capsules are white matter regions that are formed by projection fibers and white matter tracts, functioning as conduit brain regions [70,71]. Additional crucial fiber pathways and fasciculi across white matter have been previously described [65].

##### 3.1.3.2 Weeks 3–7 of gestation in the pig

Around PCD 21, the pig embryo is in the early stage of preplate formation and mRNA expression is detected at very low levels across the cerebral cortex, which continually increases until PCD 60 [72]. Early cortical formation is marked by the presence of EOMES+ and RELN+ cells at the pial surface of the cortex [32]. By PCD 23, these genes are co-expressed in certain cells, and TBR1+ cells emerge, which express TBR1, a protein important for brain development [32]. Additionally, the neural epithelium and ventricular zone are detectable in the developing pig embryo at this time [32]. At PCD 26, a striothalamic bundle penetrates the center of the developing basal ganglia area, presently a striatal mass, and is later reinforced by cortical fibers to become the internal capsule* [42]. Additionally, in the cortex, EMOES+/PAX6+ and EOMES+/PAX6- cells are present at an equal ratio, a possible indication of increased neurogenesis and basal progenitor cell genesis [32,73]. By PCD 28, while the cerebral cortex is still isolated, the anterior commissure and external capsule appear* [42,72]. At this time, the olfactory system is considered near complete, with a medial olfactory tract leading to the septal nuclei* [42]. By PCD 30, the cortical plate forms as a thin layer containing TBR1+ neurons, which indicates the onset of cortical neurogenesis, specifically within the deep cortical Layers 5 and 6* [74]. At PCD 33, above the ventricular zone, two distinct layers become evident with an inner subventricular zone containing an equal ratio of EOMES+/PAX6+ and EOMES+/PAX6- cells [32]. Furthermore, RELN+/TBR1+ cells are detectable at this time, which signify the migration and maturation of Cajal–Retzius cells that regulate cortical lamination [32]. Around PCD 35, the first trace of neuropeptide Y-immunoreactive neurons (NPY-ir) appear along the cerebral meningeal blood vessels [75]. NPY-ir cells are the most commonly distributed neuropeptides across the central nervous system, which act as modulators in a plethora of roles from neuroendocrine activity to memory function [76]. Between PCD 41 and 50, neurogenesis begins in the upper cortical Layers 2–4* [74]. At PCD 47, crossing callosal fibers become visible in front of the anterior commissure, while the caudal-most fibers of the corpus callosum appear near the frontal pole of the hemisphere, marking the corpus callosum's first appearance* [77]. The first parts of the corpus callosum to develop are the cephalic portion of the truncus, genu, and rostrum [77].

##### 3.1.3.3 Weeks 8–10 of gestation in the pig

By PCD 50, deep cortical layers no longer increase in size indicating that neurogenesis and the formation of Layers 5–6 are largely complete* [74]. At the same time, the earliest indication of formation in cortical Layers 2–4 become apparent* [74]. The ventricular zone peaks and OLIG2+ cells, specific to oligodendrocytes, are detectable in the subplate [32]. At PCD 60, peak Reelin expression is reached in the cerebral cortex, indicating a major influx of neuronal migration into the cortical plate, which will form cortical Layers 2–6 [72]. Additionally, small immunoreactive cells, which may eventually become future interneurons, are present in Layers 3–5 [72]. Within the marginal zone, the precursor of cortical Layer 1 [78], Reelin positive cells can be identified in the outer portion. Numerous growth cone bearing horizontal cells are also present, and most NPY-ir neurons reside here, which primarily remain immature [72,75]. Across cortical tissue, major sulci become recognizable and plateau levels of mRNA expression are reached with higher expression of genes involved in brain convolution such as DCX, Dab1, CDK5, and others [75,79]. At this time, the corpus callosum remains confined to the anterior portion of the brain near the frontal lobe and the splenium consists of two distinct superimposed groups of fibers [77]. The caudal extremity of the corpus callosum is coextensive with the hippocampal commissure [77]. By PCD 65, this caudal extremity extends into the superior quadrant of the brain with post-callosal and sub-callosal flexures now evident [77].

At PCD 70, upper cortical Layers 2–4 have stopped increasing in thickness, suggesting that neurogenesis has ceased* [74]. Within Layer 5, large pyramidal neurons become detectable, 2.5% of neurons are in the subplate and white matter is NPY-ir specific, with 36.5% of these cells still immature [75]. These neurons are larger than those in the marginal zone and gray matter, although axon initial segments, pivotal for mature neurons to fire action potentials, are present in both regions [75]. Additionally, by this time, the cruciate sulcus is formed [75].

##### 3.1.3.4 Weeks 11–15 of gestation in the pig

By PCD 80, the number of Reelin-positive interneurons across the cortical plate begins to decrease [72]. Additionally, cortical tissues exhibit increased expression of miR-204 and miR-34c genes that have a role in neuronal migration and neural cell differentiation, respectively [80]. There is also higher mRNA expression of genes related to calcium binding and cytoskeleton organization and biogenesis within the cortical tissue [79]. Between PCD 70 and 85, gray matter and white matter are relatively equally populated by numerous NPY-ir neurons [75]. At PCD 85, 1.7% of the neurons in the subplate and white matter are NPY-ir, with 23.6% of those cells remaining immature [75]. This decreases to 0.8%, with 8.9% of the NPY-ir cells immature by PCD 100 [75]. At this time, neurons between the cortical layers and subcortical tissue in the brain project horizontally and resemble basket cells [75]. Between PCD 80 and 115, immunoreactive cells and mRNA levels decrease across the cortex, marking the maturation and stabilization of cortical structures [72].

#### 3.1.4 Limbic system

One of the oldest systems in the brain, the limbic system, is integral for learning, memory, behavior, and emotions [81]. Similar to other mammals, pigs have been observed to exhibit a variety of social, emotional, and motivational behaviors that are driven by a complicated system of subcortical and cortical regions residing within the limbic system, including the hippocampus, fornix, thalamus, and amygdala [81,82]. Given the central role of the hippocampus in this review, focused attention will be dedicated to this region and related structures.

##### 3.1.4.1 Relevant anatomy and overview

A fundamental region of the limbic system is the hippocampus, widely recognized for its role in episodic memory, the ability to recall past experiences, by combining spatial memory with a mammal’s individual experience [83]. The main cortical input for the hippocampus is the entorhinal cortex, which is split into lateral and medial subdivisions, that provide information on the content and context of an item or event [83]. Together, these areas project to the dentate gyrus, CA1, and CA3 subregions of the hippocampus, while the deep layers of the entorhinal cortex receive feedback from the hippocampus, completing a loop [83].

The hippocampus itself is divided into multiple subdivisions and subfields, each with unique cytoarchitecture and functions. The dentate gyrus and subiculum are two of these subfields, responsible for connectivity throughout the hippocampus [84]. The subiculum also connects with the fornix, a white matter tract that supports cognitive function through sensory signals that project to the mammillary body of the hypothalamus [85]. From here, sensory projections travel through the mammillothalamic tract to complete a full feedback loop by outputting to the thalamus [85]. This circuit has an integral role in cognitive performance and memory function.

##### 3.1.4.2 Limbic system development during pig gestation

During pig gestation, the first hippocampus-related region to exhibit observable neurogenesis is the entorhinal cortex at PCD 26 [32]. Approximately a week later, on PCD 34, the first trace of the fornix appears, although its connection with the mammillary body does not emerge until PCD 40* [31]. By PCD 43, the hippocampal commissure is well developed, and its fibers extend into the lamina terminalis, a gray matter region near the third ventricle and hypothalamus that is important for homeostasis [77,86]. Here some of the fibers intersect, while another group extends further downwards and forms the anterior portion of the fornix [77]. By PCD 50, within the ventral telencephalon, prominent and large soma of entorhinal-like cells become observable while oligodendrocyte precursors are detectable in the subplate [32]. At the same time, a population of T-cell precursor cells emerges in the superficial layer of the entorhinal cortex [32]. By PCD 60, the entorhinal cortex has transitioned from having three layers to six layers, resembling the adult entorhinal cortex structure [32]. The lamina dissecans, a sparse cellular layer in the entorhinal cortex, becomes visible, dividing it into lateral and medial subdivisions along with deep-layer cells [32]. Oligodendrocyte precursors are now relatively evenly dispersed across the layers, with a higher density in the lateral entorhinal cortex, and several cells are beginning to express RELN, an encoder gene for Reelin [32]. In the hippocampus, all the flexures, or folds, are now well defined and hippocampal subdivisions, such as the presubiculum, subiculum and dentate gyrus, and white matter areas, such as the alveus and fimbria, are clearly identifiable* [77]. By PCD 70, cells in the superficial medial entorhinal cortex have become larger with more darkly stained nuclei and the entorhinal cortex shows a higher growth rate compared with the neocortex as a whole [32]. In general, neurogenesis in the entorhinal cortex concludes shortly after PCD 70, suggesting a peak in development for this region* [32]. By PCD 100, the lateral and medial subdivisions of the entorhinal cortex are clearly defined, with the lamina dissecans showing distinct features in each subdivision [32]. In the lateral portion, the lamina dissecans is diffusive with cellular infiltration, while in the medial portion, it is distinct and acellular [32]. Additionally, at this point, cresyl violet staining reveals that the cytoarchitecture of the entorhinal cortex now resembles that of a mature adult [32].

### 3.2 Species score optimization

After the literature review, a total of 30 individual neurodevelopmental events were selected for inclusion into the final model for species score calculation in the domesticated pig. These events, along with the pig species scores calculated using only the PCD of that event and assigned CLs are pictured on the pig neurodevelopmental timeline in Figure 2. Based on the CL criteria described in Tables 1 and 2, 4 events were assigned a CL of 1, 5 events a CL of 2, leaving 21 events with a CL of 3, or of high confidence in the accuracy of these values.

Individual species scores for each event were calculated using the method described in Section 2.3, resulting in a range of \[1.5\le {S}_{{\mathrm{c}}}\le 3.0]\] , which determined the candidate species score for both optimization methods described in Section 2.4. This range, coupled with a step size of 0.0001, resulted in 1,701 candidate values. We initialized the species score at 1.5 and 3.0, representing the lower and upper bounds of the predefined range.

**Figure 3:**
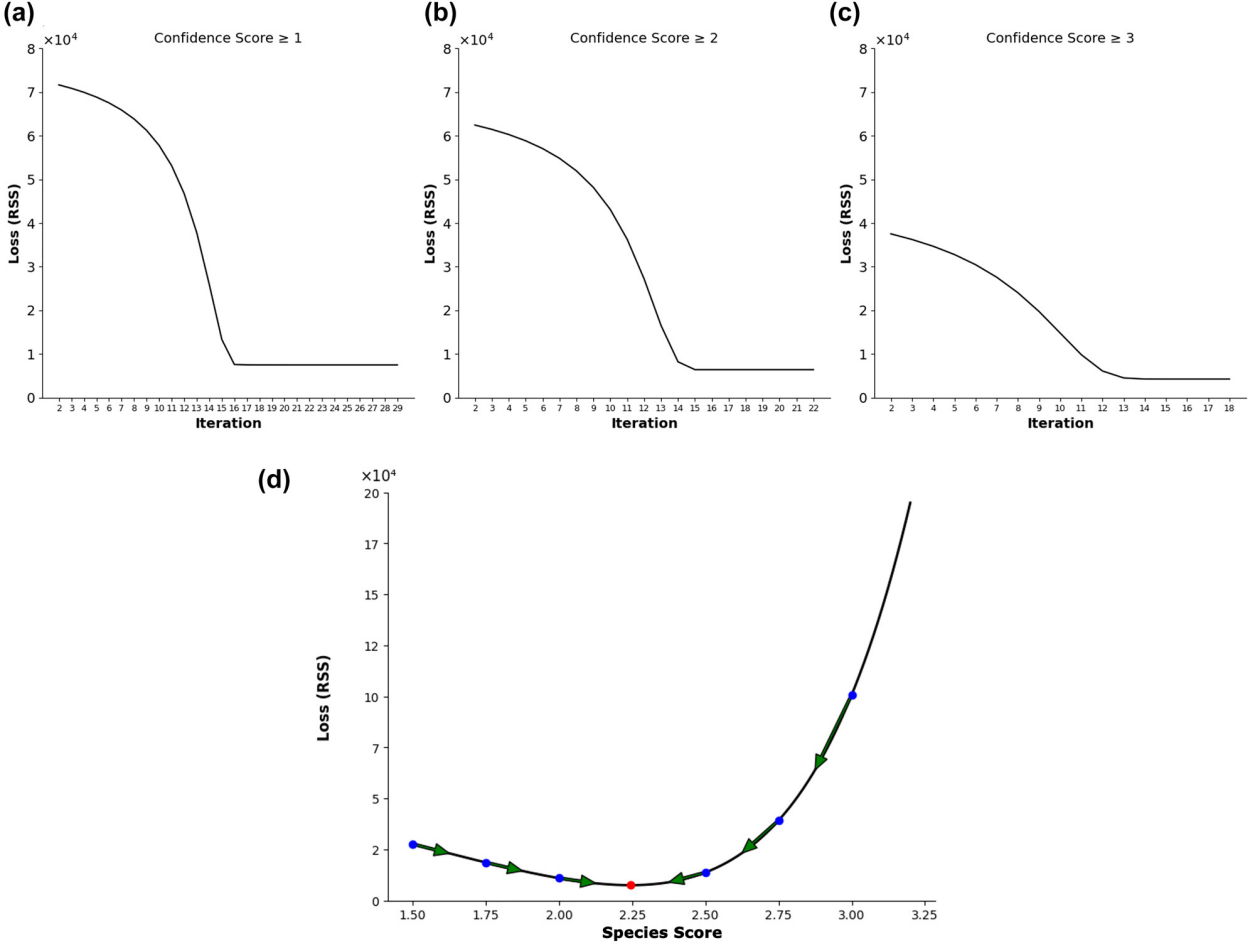
Species score optimization with gradient descent. Graphs (a)–(c) correspond to the minimization of RSS loss utilizing the gradient descent method. With each iteration, events were omitted based on assigned CLs. In the first iteration (a), all events were included, resulting in an RSS of 7509.45. The second iteration (b) only included events with \[{\mathrm{CL}}\ge 2]\] , resulting in an RSS of 6424.51. The third iteration (c) only included events with a \[{\mathrm{CL}}\ge 3]\] , resulting in an RSS of 4260.46. Graph (d) depicts the step-by-step learning rate used with gradient descent to arrive at the pig species score of 2.157 (red point) with the smallest cost function.

**Figure 4:**
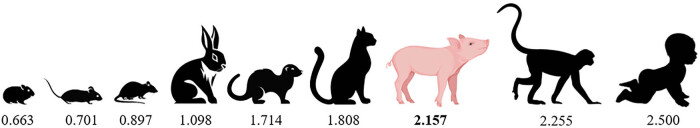
Pig species placement in the translating time model. This figure depicts the positioning of the domestic pig in the translating time model created by Clancy et al. [21]. The pig was determined to have a species score of 2.157, placing it right between the cat (1.808) and the macaque (2.255). At the lowest end of the model sits the hamster (0.663), followed by the mouse (0.701), rat (0.897), rabbit (1.098), spiny mouse (1.177, not pictured), and the ferret (1.714). At the highest end after the macaque is the human infant with a species score of 2.500.

Using the grid search optimization method, including all events resulted in a species score of 2.244 with an RSS of 7509.45. Repeating the same method but taking into account events with a \[{\mathrm{CL}}\ge 2]\] , resulted in a species score of 2.232 and an RSS of 6424.51. Finally, with the last repetition, only events with a \[{\mathrm{CL}}=3]\] were included, resulting in a species score of 2.157 and a reduced RSS of 4260.47. The gradient descent method produced nearly identical results (Figure 3). Including all events resulted in a species score of 2.244 and an RSS of 7509.45. Including events with a \[{\mathrm{CL}}\ge 2]\] resulted in a species score of 2.232, an RSS of 6424.51. Finally, including events with a \[{\mathrm{CL}}=3]\] , resulted in a species score of 2.157, and a reduced RSS of 4260.46. Across both optimization methods, the domestic pig was consistently determined to have a species score of 2.157, positioning it right between the cat and macaque in the translating time model (Figure 4). Using this selected species score, the predicted PCDs for all 95 neurodevelopmental events from Clancy et al. [21] are modeled out in Table S1.

### 3.3 Domestic pig species score placement

The domestic pig's placement in the translating time model positions it closer to human infants in terms of neurodevelopment. In the Clancy et al. [21] translating time model, a species score closer to 0 indicates that this species’ neurodevelopment is comparable to rodents such as the hamster, mouse, and rat. Conversely, scores closer to 2.5 correspond to a neurodevelopment trajectory comparable with primate models such as human and macaque. The placement of the domestic pig between the cat and macaque is supported by biological evidence from our comprehensive literature search.

Regarding anatomical structure, the pig brain resembles the elongated oval shape of the monkey brain [12]. However, there are several key differences such as a less pronounced telencephalon, less developed anterior pole, and much larger and pronounced olfactory bulbs [12]. Compared with cats, the adult pig brain is almost triple the size (80–180 g) and contains relatively more neocortical neurons (325–430 million) [87,88]. Additionally, the pig’s relative brain-to-body weight ratio is closer to primates compared with the cat [88], further reinforcing our placement of the domesticated pig in the translating time model. Pigs also exhibit a perinatal brain growth spurt similar to humans and reach around 25% of their adult brain weight by birth [89]. Additionally, the pig brain's gray-to-white matter ratio (60% white matter) mirrors that of humans [90]. These similarities have already driven researchers to utilize the pig to model neurological disorders as well as the impacts of early life nutrition and stressors on brain development [8,9,87,90].

In terms of visual development, pigs are closer to primates than cats or rodents [47]. Cats, like rodents, are born with an immature visual system and open their eyes around 7–10 days after birth [91]. On the other hand, pigs are born with a relatively well-developed visual system and open their eyes at birth or soon after [47,91]. In humans and non-human primates, visual development primarily occurs prenatally, and eyes can respond to light by the seventh month of gestation [47].

Despite these similarities, several key factors contribute to the placement of the pig after the primate. First, pigs are relatively precocial, because they are born with open eyes and independent locomotion [48]. In contrast, more altricial species, such as rodents, cats, and primates, rely heavily on parental care because they are born at a relatively earlier stage of development with limited motor capabilities [92]. Pigs, while precocial, still rely heavily on maternal care early in life, particularly for the provision of milk [92,93]. Other distinctions include the pig’s shorter gestational length (114–116 days) and large litter size [23,94]. These two aspects are crucial as differences in the size of the neonatal brain correlate with gestational length and litter size [94]. Therefore, since primates and humans, have a gestational period (∼280 days) more than double that of the pig and typically bear a single offspring, it aligns with them having the largest relative brain size among mammals [88,94]. Considering gestational length, litter size, and neonatal brain size, the placement of the pig between the non-human primate and cat would be expected.

### 3.4 Limitations

Although the following model and derived domestic pig species placement is well supported by the literature, there are some notable limitations to consider. When defining the inclusion and exclusion criteria, the calculated species score was sensitive to the collection of neurodevelopmental events that were included. In several instances, a stricter approach was taken, which caused the derived species score to become biologically irrelevant. For example, an approach was taken where neurodevelopmental events were excluded when the event was derived from purely an anatomical assessment or when the literature was considered outdated ( \[< 1990{\mathrm{s}}]\] ). Removing these events resulted in 10 or fewer events with a \[{\mathrm{CL}}=3]\] , which pushed the pig’s species score to 2.3 or higher placing it above the macaque (species score = 2.255). Based on the previous species placement discussion, this does not follow the expected biological placement since primates are the closest species to humans when considering brain size and development [88]. The decision was made to use a more robust approach such that events with higher confidence were not given inappropriate weighting in the model. Additionally, taking a more robust approach minimized the loss from an RSS of approximately 7,000 to 4,000, further reinforcing that this was the most appropriate approach.

The potential reason for the sensitivity of our current model can be attributed to the original data. Clancy and colleagues [21] included data across 9 different mammalian species resulting in a total of 362 observations derived from literature. With this large subset of data, they tailored their statistical model to take a cross-species approach whereas in our instance we used the pre-existing model for including the domestic pig as a new species. Additionally, the various neurodevelopmental events that were chosen for the pig were not from the same literature base as used by Clancy et al. [21], who primarily relied on pre-existing reports of neurogenesis counts across the selected animal species. This variance could also impact the sensitivity of the model since the same reports were not available for the domestic pig. Nevertheless, to the best of our knowledge, this remains the best and only established method for translational comparison across species and represents the first attempt to include the domestic pig.

Although utilizing the domestic pig as a biomedical model has grown over recent decades, there are many avenues of research that require further exploration and consolidation. In the cognitive and behavioral fields, multiple behavioral paradigms such as classical conditioning, operant conditioning, spatial learning, and memory tasks have been successfully modeled in the pig [95]. Additionally, pigs have been observed to exhibit social recognition, respond to antipsychotic drugs, and develop depression-like symptoms after chronic stress [95]. However, interpretation of behavioral data can be difficult to discern due to inconsistencies in methods, reporting idiosyncrasies, and overall limited validation of techniques. Recently, researchers have re-evaluated and established guidelines for conducting behavioral paradigms such as novel object recognition [96], open field test, elevated plus maze, and other paradigms, but in general, interpretation remains ambiguous due to a lack of standardization in behavioral testing design, execution, and analysis [97]. Additionally, progress has been made in developing transgenic pig models to study various human diseases such as muscle disorders, cancers, dementia, and neurodevelopmental disorders [10,98,99]. Nevertheless, compared with rodent work, transgenic work in the pig is still in its infancy and requires further refinement. Therefore, future work should focus on investigating these various discrepancies, refining methods, and establishing consolidated protocols to further advance the utilization of the pig as a translational animal model.

## 4 Conclusion

Currently, in the field of translational animal model research, a comprehensive review of the pig prenatal neurodevelopmental timeline has been notably absent, despite the pig’s potential as a valuable animal for neurodevelopmental research. While the domestic pig has already been extensively used as a biomedical animal model for humans, a clear framework for translating neurodevelopmental time between these species has been ambiguous. The current review aimed to tackle both issues. After conducting a thorough review of the literature, we compiled a detailed breakdown of neurodevelopment from week three of gestation until birth. From here, a total of 30 neurodevelopmental events were selected to include in the translating time model established by Clancy and colleagues [21]. The analysis determined that the domestic pig is positioned between the cat and macaque on the neurodevelopmental translational timeline. In conclusion, this review establishes the domestic pig as a highly relevant model for studying human neurodevelopment, second only to primates.

## Supplementary Material

Supplementary Table
