# Supplementary material for: Establishing the pig as a translational animal model for neurodevelopment
Source: Transl Neurosci. 2025 Apr 24;16(1):20250369. doi: 10.1515/tnsci-2025-0369 (PMC12032983; doi:10.1515/tnsci-2025-0369)
Supplement: Supplementary Table [file tnsci-2025-0369-sm.pdf]

Supplementary material

Table S1: Predicted PCDs for translating mammalian time neurodevelopmental events[1]

Neurodevelopmental event	Event score	Pig predicted PCD
Cranial motor nuclei - peak	0.903	25.7
Retinal ganglion cell generation - start	1.023	28.5
Subplate - start	1.038	28.8
Locus coeruleus - peak	1.070	29.6
Inferior olivary nucleus - peak	1.073	29.7
Magnocellular basal forebrain - peak	1.103	30.5
Superficial SC laminae - start	1.126	31.1
Posterior commissure appears	1.126	31.1
Red nucleus - peak	1.128	31.1
Vestibular nuclei - peak	1.128	31.1
Cranial sensory nuclei - peak	1.155	31.8
dLGN - start	1.156	31.9
External capsule appears	1.163	32.1
Subplate - peak	1.172	32.3
Reticular nuclei -peak	1.200	33.1
Medial geniculate nucleus - peak	1.233	34.1
Raphe complex - peak	1.237	34.2
Cortical layer VI - start	1.244	34.4
Mammillothalamic tract appears	1.248	34.5
Axons in optic stalk	1.250	34.6
Purkinje cells - peak	1.251	34.6
Deep cerebellar nuclei - peak	1.256	34.8
Preoptic nucleus - peak	1.258	34.8
Globus pallidus - peak	1.272	35.3
vLGN - peak	1.276	35.4
Medial forebrain bundle appears	1.302	36.2
Internal capsule appears	1.305	36.3
dLGN - peak	1.323	36.9
Suprachiasmatic nucleus - peak	1.330	37.1
Fasciculus retroflexus appears	1.338	37.4

Table S1: *Continued*

Neurodevelopmental event	Event score	Pig predicted PCD
Optic axons at chiasm of optic tract	1.342	37.5
Cochlear nuclei - peak	1.343	37.5
Rapid axon generation/optic nerve - start	1.366	38.3
Mitral cells - peak	1.373	38.5
VP and VB nuclei - peak	1.375	38.6
Nucleus of lateral olfactory tract - peak	1.375	38.6
Retinal horizontal cells - peak	1.375	38.6
Amygdala - peak	1.392	39.2
Superior colliculus - peak	1.404	39.6
Claustrum - peak	1.410	39.8
Stria medullaris thalami appears	1.414	40.0
dLGN - end	1.416	40.0
Substantia nigra - peak	1.420	40.2
Entorhinal cortex - peak	1.421	40.2
Cortical layer V - start	1.422	40.2
Retinal ganglion cells - peak	1.439	40.9
Anterior olfactory nucleus - peak	1.441	40.9
Subplate - end	1.442	41.0
Cortical layer VI peak	1.448	41.2
Septal nuclei - peak	1.463	41.7
Inferior colliculus - peak	1.475	42.2
AV, AM and AD nuclei - peak	1.483	42.5
Optic axons reach dLGN and SC	1.519	43.9
Pontine nuclei - peak	1.528	44.3
Caudoputamen - peak	1.532	44.4
Optic axons invade visual centers	1.542	44.8
Subiculum - peak	1.545	44.9
Parasubiculum - peak	1.556	45.4
Superficial SC laminae - end	1.571	46.0
Fornix appears	1.573	46.1
Stria terminalis appears	1.578	46.3

(Continued)

Table S1: *Continued*

Neurodevelopmental event	Event score	Pig predicted PCD
Cortical layer V - peak	1.582	46.5
Presubiculum - peak	1.583	46.5
Cortical lamina VI-end	1.596	47.1
Cortical lamina IV - start	1.607	47.5
Dentate gyrus - peak	1.617	48.0
Anterior commissure appears	1.620	48.1
Cones - peak	1.630	48.5
CA 1, CA 2-peak	1.637	48.8
Retinal amacrine cells - peak	1.678	50.7
Cortical layer II/III - start	1.681	50.8
Cortical layer V-end	1.689	51.2
Nucleus accumbens - peak	1.690	51.3
Tufted cells - peak	1.691	51.3
Cortical layer IV-peak	1.703	51.9
Hippocampal commissure appears	1.711	52.2
Retinal ganglion cell generation - end	1.725	52.9
Corpus callosum appears	1.742	53.8
Isles of Calleja - peak	1.748	54.1
LGN axons in subplate	1.768	55.1
Cortical axons reach dLGN	1.794	56.4
Cortical layer II/III - peak	1.826	58.1
Cortical layer IV - end	1.839	58.8

Table S1: *Continued*

Neurodevelopmental event	Event score	Pig predicted PCD
Optic nerve axon number -peak	1.849	59.3
Cortical layer II/III - end	1.929	63.9
Cortical axons innervate dLGN	2.005	68.6
Adult like cortical innervation of dLGN	2.103	75.2
LGN axons in cortical layer IV	2.117	76.2
Superficial SC - start of lamination	2.124	76.7
Rods - peak	2.136	77.6
Visual cortical axons in SC	2.212	83.4
Retinal bipolar cells - peak	2.215	83.6
Ipsi/contra segregation in LGN and SC	2.298	90.4
Rapid axon loss in optic nerve ends	2.331	93.3
Eye opening	2.546	114.7

[1]All neurodevelopmental events and events scores were directly pulled from the Clancy et al. [19] model. The pig predicted PCDs were calculated using the Clancy et al. [19] equation and the optimized pig species score of 2.157. Abbreviations: AD, anterior dorsal thalamus; AM, anteromedial thalamus; AV, anteroventral thalamus; dLGN, dorsal lateral geniculate nucleus; vLGN, ventral lateral geniculate nucleus; SC, superior colliculus; VB, ventrobasal thalamus; VP ventroposterial thalamus; PCD, postconception day.
